# Treatment of Chronic Degenerative Hip Pain in a Male Patient With Cerebral Palsy Four Decades After Reverse Osteotomy

**DOI:** 10.7759/cureus.46495

**Published:** 2023-10-04

**Authors:** John B Hooks, David Dayya

**Affiliations:** 1 Medicine, College of Osteopathic Medicine, University of New England, Biddeford, USA; 2 Family Medicine, New England Healthcare, New Canaan, USA

**Keywords:** iliopsoas bursitis, osteoarthritis, hip subluxation, gait abnormalities, coxa valga, reverse osteotomy, orif, cerebral palsy

## Abstract

Patients with cerebral palsy (CP) frequently require surgical hip interventions in early adulthood due to spasticity-related gait abnormalities. In most instances, these cases are characterized by severe restrictions on mobility. This is the case of a male patient with CP who underwent right proximal femoral open reduction internal fixation (ORIF) and reverse osteotomy for right hip subluxation in young adulthood. Patients with CP who undergo total hip arthroplasty (THA) or ORIF with reverse osteotomy often require future revision. The patient was initially given an estimated 10-year longevity for his plate and screw construct (hardware). Forty-four years later, the patient presented with debilitating chronic bilateral hip pain, requiring the assistance of a cane for ambulation. There is a limited body of knowledge on ORIF and reverse osteotomy follow-up in patients with CP within a 30- to 50-year period. At the 44th-year follow-up, CT and X-ray imaging found postoperative changes in the right femur, including intact hardware, bilateral acetabular dysplasia, right femoral stress fracture, progression of hip arthritis, and right iliopsoas bursitis. Surgery for hardware revision was not indicated. Gradual restoration of function was achieved over a 14-month period with conservative management. This case suggests that physical therapy (PT), exercise, and sporadic non-steroidal anti-inflammatory drug (NSAID) use are effective for improving chronic degenerative changes, associated bursitis, and loss of function in patients who developed CP-induced gait complications in young adulthood. These improvements can be made several decades after undergoing ORIF and osteotomies. This course of treatment was effective in improving the patient’s quality of life without additional surgical interventions.

## Introduction

Cerebral palsy (CP) results in gait disturbances that often require surgical intervention in young adulthood [1]. Patients with CP frequently have a reduced range of motion at the hip joint due to contractures and spasticity [2]. Most commonly, CP leads to hip dysplasia from weakness of abductor muscles and spasticity of hip flexor and adductor muscles, causing a delay in weight bearing. This leads to deformities of the proximal femur and progressive dysplasia of the acetabulum [2]. Complications often occurring early in adulthood include a risk for subluxation from the coxa valga, increased femoral anteversion, and associated muscle imbalances [3]. Surgical repair was traditionally indicated in early adulthood with osteotomy and proximal femoral fixation, although total hip arthroplasty (THA) has now become more commonplace [1]. There are few published case studies on the long-term outcomes of post-surgical patients for periods greater than 10 years [4].

Late-adulthood chronic hip pain and associated gait disturbances in patients with CP can be treated by conservative management, including bracing, botulinum toxin A injections, and heel lifts. If these are not sufficient to improve gait and quality of life, surgical reconstruction or revision of prior hardware may be required [3,5]. The patient in this case experienced chronic hip pain and gait disturbances 44 years after the initial surgical intervention. The case presented challenges since the patient lacked a strong indication for aggressive surgical intervention and was unable to control pain with pharmacological intervention alone.

This article was previously presented as a meeting abstract at the 2023 Northeast Osteopathic Medical Education Network (NEOMEN) Annual Research and Scholarship Forum.

## Case presentation

**Figure 1:**
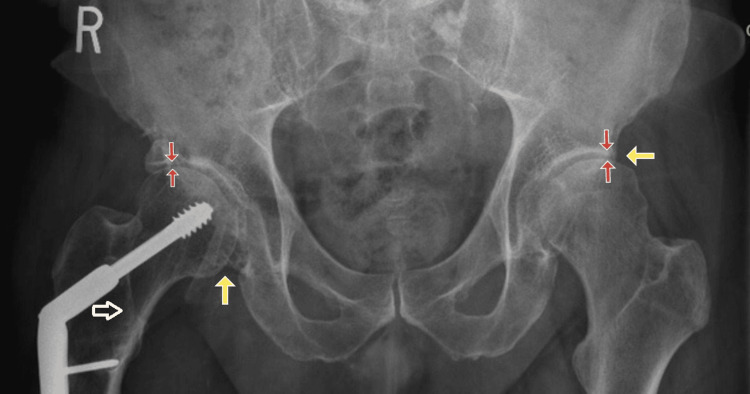
Bilateral hip X-ray The X-ray imaging shows postoperative changes of the right femur with bilateral acetabular dysplasia (yellow arrows), a healed right intertrochanteric stress fracture (white arrow), and progression of bilateral coxarthrosis (red arrows).

A 64-year-old male with a past medical history of CP presented to his primary care physician with bilateral hip pain. At age 20, CP-related spasticity resulted in a right hip subluxation, which was repaired with ORIF and a reverse osteotomy. The patient had been fully ambulatory for the subsequent 44 years with a residual limp gait, right heel lift for short leg syndrome, and mild chronic right hip pain rated 2/10 that did not affect his daily activities. The patient reported a fall five months prior, during which he landed on his right side and experienced aggravation of his baseline chronic right hip pain. Before the fall, he was able to exercise regularly, which included walking, an elliptical machine, and strength training. At the time of presentation, he weighed 74.3 kg, and his body mass index was 25.6 kg/m2. Over the course of two months, he became unable to bear weight and required caregiver assistance. The patient reported 10/10 intractable pain in his left hip and 5/10 pain in his right hip. He was unable to sit comfortably without pain for an extended period of time. The pain was not alleviated with ice, heat, or over-the-counter analgesics. There was no pattern of pain in relation to the time of day. Four days before the initial presentation, the patient underwent an X-ray of the hips bilaterally, which demonstrated postoperative ORIF of the right femur with bilateral acetabular dysplasia, a healed right femoral stress fracture, and progression of right hip arthritis (Figure 1). The patient was sent to the ER for further evaluation and an emergent CT of his bilateral hips as he was deemed a high-fall risk, and the case was complicated by pre-existing hardware.

In the ER, a physical exam revealed decreased left straight leg raising compared to the right, which induced pain in the lateral hip and proximal femur. Pain in this location was additionally elicited with abduction and adduction. Exam findings included a baseline leg length discrepancy, with the right leg being shorter than the left. Straight leg raising of the right did not induce pain or sciatica, nor did adduction or abduction of the right leg. The pelvis was non-tender. The general examination was unremarkable.

**Figure 2:**
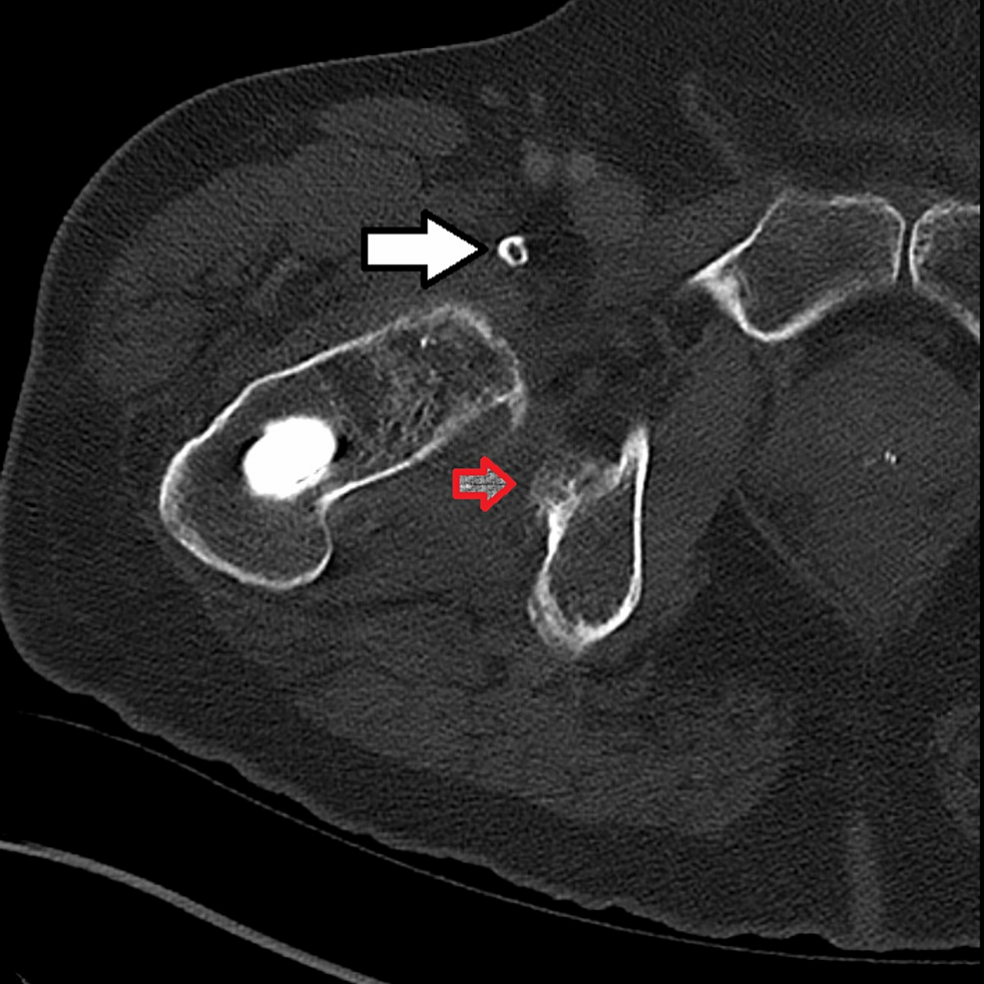
CT image of the right hip, axial view The right hip shows evidence of a 3.2 x 1.4 x 1.2 cm well-corticated ossification involving the lateral acetabulum (red arrow) and calcification of the iliopsoas tendon with a 2.9 x 2.6 x 1.6 cm region of fluid anterior to the acetabulum iliopsoas bursa (white arrow).

He received CT imaging studies of both hips. The right hip CT scan with IV contrast demonstrated postoperative changes from prior ORIF and a right healed intertrochanteric fracture. There was a compression screw visualized in the right femoral head and a lateral plate and screw construct involving the proximal right femoral diaphysis. There was no acute fracture. Additional findings included a 2.9 x 2.6 x 1.6 cm region of fluid anterior to the acetabulum iliopsoas bursa, calcification of the iliopsoas tendon, and a 3.2 x 1.4 x 1.2 cm well-corticated ossification involving the lateral acetabulum (Figure 2). There was no finding of a pelvic fracture.

**Figure 3:**
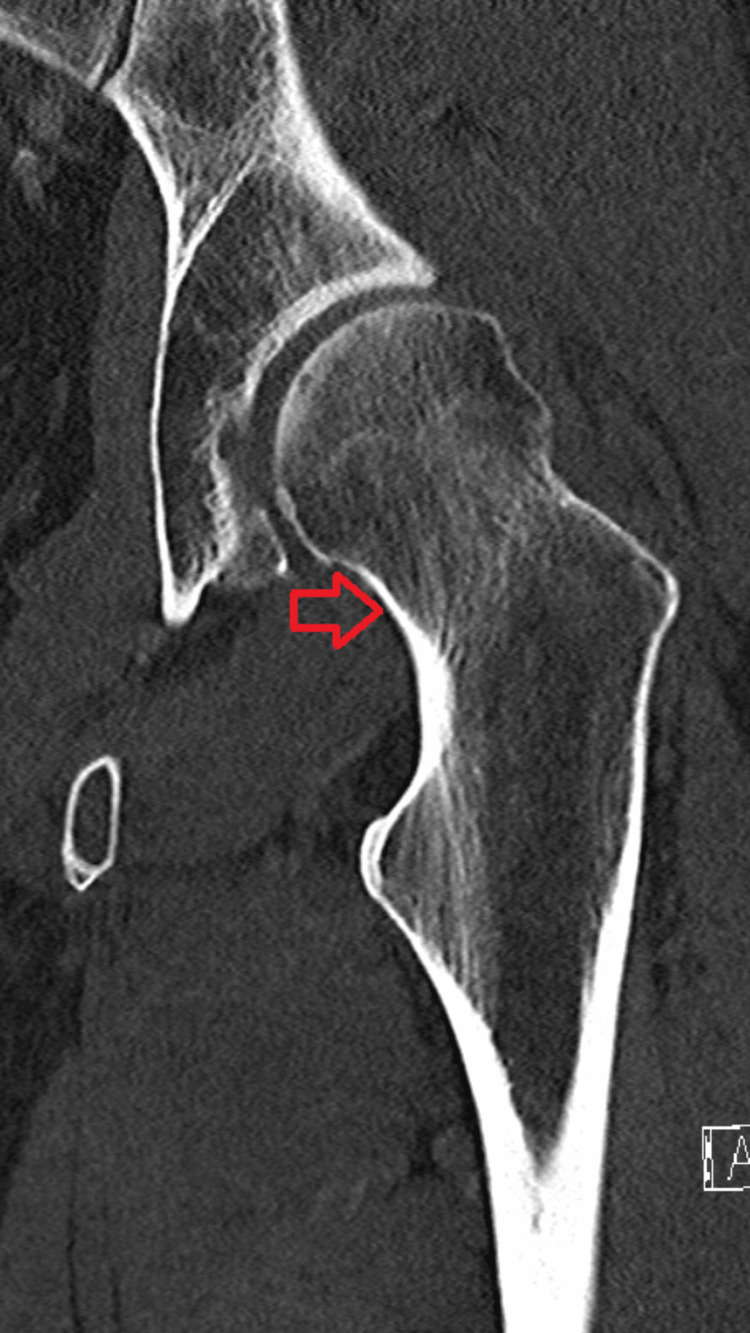
CT image of the left hip, coronal view The CT reveals partial uncovering of the left hip with evidence of dysplasia and enlargement of the femoral neck (red arrow).

The CT imaging of the left hip with IV contrast demonstrated mild to moderate loss of articular cartilage in the superior or weight-bearing surface. There were small marginal osteophytes and a partial uncovering of the left hip with evidence of dysplasia and enlargement of the femoral neck (Figure 3). The image was negative for an acute fracture of the hip, and there was no pelvic fracture visualized. The complete blood count and comprehensive metabolic panel lab studies were within normal limits.

After ER evaluation, the patient was referred to orthopedics, who evaluated the bilateral hip dysplasia and arthritic changes, including the position and integrity of the hardware in the right hip and the healed femoral stress fracture. The pain was determined to be caused by a combination of arthritic changes and bursitis. The orthopedic recommendation was to administer injectable steroids, and the patient was given a referral to physical therapy (PT). The patient declined injectable steroids, opting instead for PT, meloxicam 15 mg PO daily for three weeks, and diclofenac sodium gel application daily.

Seven months after diagnosis, the patient had not entered PT. He continued meloxicam for three months with minimal relief and reported pain ranging from 4 to 8/10 in the left hip. He remained unable to ambulate without assistance for the entirety of the meloxicam and diclofenac sodium gel treatment. During these seven months, he was sedentary other than basic daily living activities. He continued to decline steroid injections. The patient began a home resistance band training program, which reduced pain to 4/10 bilaterally consistently. The range of motion improved within one month, and the patient was able to ambulate without assistance for distances of 10 to 15 feet.

He began a PT program at month 10, attending twice per week for one hour. The patient continued a home exercise regimen with over-the-counter non-steroidal anti-inflammatory drug (NSAID) doses as needed. The home exercise routine included the use of a recumbent stepper, an arc trainer, an elliptical trainer, a recumbent bike, and various resistance machines.

Following one month of PT, the patient was able to ambulate without pain or assistance for distances of 50 feet, with no falls reported. In the following month, he experienced occasional aggravations of pain associated with long periods of sitting in a vehicle, which were alleviated by stretching techniques and at-home exercises. The patient had been experiencing waxing and waning pain and reduced ambulation, alternating with restoration of function, since that time. With continued home strengthening and resistance techniques prescribed by PT, the patient eventually improved to ambulating distances of 200 feet without assistance. Function has been adequately restored, although the patient has not returned completely to the post-surgical baseline.

## Discussion

Patients with CP frequently have a reduced range of motion at the hip joint due to contractures and spasticity. Complications often occurring early in adulthood include a risk for hip subluxation from the coxa valga, increased femoral anteversion, and associated muscle imbalances [3]. The CP-induced subluxation and dislocation are treated surgically to prevent femoral head degeneration and secondary osteoarthritis of the acetabulum [1]. Over time, the hip joints can become incongruent, leading to the further development of arthrosis.

Coxarthrosis in patients with CP is frequently treated with THA. Prior studies of CP showed coxarthrosis treated with THA at an average age of 30.6 years was successful in restoring pre-pain function for approximately 88% of patients. This was true within a follow-up range of two to 28 years, with nine out of 56 patients requiring revisions [3]. The maximum average post-surgical follow-up for any existing study was 10 years [1]. The mean age at the time of surgery was 42.0 years [6].

The mild nature of this patient’s CP ruled out THA as it would limit his physical activity participation in young adulthood. His increased mobility due to the relatively mild nature of his CP is relatively uncommon in this patient population. This may explain the limited body of knowledge on ORIF and reverse osteotomy follow-up in patients with CP within a 30- to 50-year period.

Proximal femoral subluxation, which was the primary indication for this patient’s surgical intervention in young adulthood, has an incidence as high as 79% [3]. Proximal femoral deformities are most often attributed to delayed weight bearing due to a CP-induced gait characterized by abductor weakness coinciding with hip adductor and flexor spasticity [2]. Therefore, PT and strengthening exercises are beneficial to a patient with CP at any stage of life. The patient in this case had not participated in any PT since the time of his initial hardware placement. Hip subluxation in patients with CP is measured by the migration percentage (MP). This patient’s CP was classified as the spastic subtype, which often presents with subluxation at an earlier age than other CP subtypes [5].

A 2019 meta-analysis by Agarwal et al. determined that the resubluxation and reoperation rate for femoral osteotomy as performed on this patient is 22.9%. There were 51% decreased odds of resubluxation in patients who underwent a combined femoral and pelvic osteotomy as opposed to a femoral osteotomy alone, as in this patient’s case. The longest average follow-up time for any prior study was 19 years [4]. At the time of this patient’s presentation, he had doubly exceeded that time frame and still did not require reoperation.

Patients with CP have a high rate of comorbid chronic disease development. This is especially high in the CP patient population as they enter middle age. Osteoarthritis due to gait abnormalities is a common comorbidity in this population. Restricted mobility due to gait abnormalities is a factor that contributes to obesity, which then further increases the likelihood of developing musculoskeletal and cardiovascular comorbidities [7]. The patient in this case was considered overweight but not obese. The severity of osteoarthritis-associated hip pain is not consistently correlated with radiographic findings. It is not uncommon for patients with findings of mild to moderate arthritic changes on X-ray imaging to experience severe pain, or for patients with findings of severe arthritis on radiographs to report only moderate pain [8]. The patient in this case is an example of the former. The early onset of osteoarthritis and other chronic diseases in adolescent CP patients is a predictor of an increased likelihood of disease acceleration in adulthood [9]. Patients with CP are also susceptible to the same personal-level risk factors for hip osteoarthritis as those in the general population, which may exacerbate the gait-induced deformities of their hip joint. These factors include advancing age, diet, female gender, increased weight, and genetics [10].

The patient in this case declined all pharmacological injections into the hip joint. Intra-articular corticosteroid injections are the most common injectable agent offered for pain relief. Other options include hyaluronic acid, NSAIDs, local anesthetics, or a combination of these. The most common combinations are triamcinolone or methylprednisolone mixed with 1% lidocaine or 0.5% bupivacaine. It is recommended that these injections avoid being repeated, and there must be a three-month period between the injection and a THA procedure [11]. The successful rehabilitation of the patient in this case without the use of intra-articular injections may be attributed in part to consistent physical activity in childhood. It is well documented that CP patients who are able to participate in consistent moderate-intensity physical activity in childhood and adolescence have a greater chance of lifelong mobility [12].

A 2021 cohort study by van Berkel et al. found that within 10 years of the initial complaint of osteoarthritic hip pain, 12% of patients underwent hip replacement, and the remainder reported stabilization of symptoms or fewer clinical complaints. Among those who did not undergo hip replacement, pain medication use only increased from 43% at baseline to 50% at 10-year follow-up [13]. Therefore, it is likely that the long-term follow-up of the patient in this case may reveal continued stabilization of symptoms, with hip replacement as a less likely outcome. However, this study was not exclusive to patients with CP.

## Conclusions

The treatment of chronic degenerative changes of the hip over four decades after ORIF and reverse osteotomies in patients with CP has not been well studied. The patient in this case presented 44 years after the initial surgical intervention with chronic degenerative changes and severely reduced mobility. Conservative management, including PT, exercise, and sporadic NSAID use, was effective in restoring function over a 14-month period. This case demonstrates that deterioration of gait from chronic degenerative changes can be adequately reversed several decades after undergoing ORIF and reverse osteotomy for CP-induced gait disturbances, without steroid injections, THA, or ORIF revision. The patient's outcome with this treatment plan suggests that if hardware revision and steroids are not included, successful rehabilitation is still possible but may require a period of greater than one year of dedication to frequent PT treatments and home exercises.
